# Diagnostic and prognostic value of interleukin-6, pentraxin 3, and procalcitonin levels among sepsis and septic shock patients: a prospective controlled study according to the Sepsis-3 definitions

**DOI:** 10.1186/s12879-019-4618-7

**Published:** 2019-11-12

**Authors:** Juhyun Song, Dae Won Park, Sungwoo Moon, Han-Jin Cho, Jong Hak Park, Hyeri Seok, Won Seok Choi

**Affiliations:** 1 Department of Emergency Medicine, Korea University Ansan Hospital, Ansan, Republic of Korea; 2 Division of Infectious Diseases, Department of Internal Medicine, Korea University Ansan Hospital, 123, Jeokgeum-ro, Danwon-gu, Ansan-si, Gyeonggi-do Republic of Korea

**Keywords:** Interleukin-6, Pentraxin 3, Procalcitonin, Sepsis, Septic shock, Emergency department

## Abstract

**Background:**

This study investigated the clinical value of interleukin-6 (IL-6), pentraxin 3 (PTX3), and procalcitonin (PCT) in patients with sepsis and septic shock diagnosed according to the Third International Consensus Definitions for Sepsis and Septic Shock (Sepsis-3).

**Methods:**

Serum levels of IL-6, PTX3, and PCT were measured in 142 enrolled subjects (51 with sepsis, 46 with septic shock, and 45 as controls). Follow-up IL-6 and PTX3 levels were measured in patients with initial septic shock within 24 h of hospital discharge. Optimal cut-off values were determined for sepsis and septic shock, and prognostic values were evaluated.

**Results:**

Serum IL-6 levels could discriminate sepsis (area under the curve [AUC], 0.83–0.94, *P* <  0.001; cut-off value, 52.60 pg/mL, 80.4% sensitivity, 88.9% specificity) from controls and could distinguish septic shock (AUC, 0.71–0.89; cut-off value, 348.92 pg/mL, 76.1% sensitivity, 78.4% specificity) from sepsis. Twenty-eight-day mortality was significantly higher in the group with high IL-6 (≥ 348.92 pg/mL) than in the group with low IL-6 (< 348.92 pg/mL) (*P* = 0.008). IL-6 was an independent risk factor for 28-day mortality among overall patients (hazard ratio, 1.0004; 95% confidence interval, 1.0003–1.0005; *p* = 0.024). In septic shock patients, both the initial and follow-up PTX3 levels were consistently significantly higher in patients who died than in those who recovered (initial *p* = 0.004; follow-up *P* <  0.001).

**Conclusions:**

The diagnostic and prognostic value of IL-6 was superior to those of PTX3 and PCT for sepsis and septic shock.

## Background

Sepsis is an important public health issue globally. Despite advances in modern medicine, over 5.3 million people die annually from sepsis, at an estimated overall mortality of 30% [1–3]. According to the Third International Consensus Definitions for Sepsis and Septic Shock (Sepsis-3), sepsis is defined as life-threatening organ dysfunction caused by dysregulated host response to infection [1, 2, 4]. Early identification and diagnosis are essential, as prompt and appropriate treatment can improve survival outcomes [5]. Despite pre-existing diagnostic criteria, early diagnosis of sepsis is usually complex due to unknown sources of infection and vague sepsis syndrome definitions [6]. C-reactive protein (CRP) and procalcitonin (PCT) have been widely used to facilitate sepsis diagnosis, but their diagnostic and prognostic values are limited [7–11]. Improved biomarkers are therefore required for the prompt diagnosis of sepsis and prediction of outcomes.

Interleukin-6 (IL-6), a pro-inflammatory cytokine, is synthesized from T-lymphocytes, fibroblasts, endothelial cells, and monocytes [12, 13]. IL-6 serves as an important mediator during the acute phase of response to inflammation in sepsis, and its clinical value has been assessed in patients with various septic conditions in several studies [14–20]. However, the diagnostic and prognostic values of IL-6 are controversial. A recent study reported that the IL-6 level is a diagnostic marker of infection as well as a prognostic marker in patients with organ dysfunction [20]. However, a meta-analysis of diagnostic performance showed that IL-6 offers only moderate success in differentiating sepsis from non-infectious systemic inflammatory response syndrome (SIRS) in adults [12]; the use of IL-6 was thus recommended as a diagnostic aid to confirm rather than exclude infection in patients with SIRS.

Pentraxin 3 (PTX3), which belongs to the long pentraxin family, is expressed in a variety of cells during various inflammatory processes, including sepsis [21]. PTX3 plays a role in the early phase of inflammation by activating the classical complement pathway and facilitating recognition by macrophages and dendritic cells [21, 22]. Evidence concerning the clinical value of IL-6 and PTX3 is controversial; however, prior studies have proposed PTX3 as a diagnostic and prognostic marker of sepsis [23–30]. In a systematic review and meta-analysis, PTX3 was identified as a marker of sepsis severity and predictor of mortality [31]. A recent study showed that PTX3 discriminated sepsis and septic shock patients from controls in a medical intensive care unit (ICU) in accordance with the Sepsis-3 definitions [32], suggesting that PTX3 has a diagnostic value comparable to that of IL-6 in sepsis and septic shock.

The present study aimed to investigate both the diagnostic and prognostic values of IL-6, PTX3, and PCT in the emergency department (ED) patients with sepsis and septic shock using the Sepsis-3 definitions.

## Methods

### Study design

This study was a single-center prospective controlled study of sepsis patients who visited the ED of Korea University Ansan Hospital, Korea, which is an 870-bed tertiary care teaching hospital with an annual load of approximately 50,000 patients. The study was conducted from December 13, 2017 to June 5, 2018. The newly developed Intelligent Sepsis Management System (i-SMS), which employs Sepsis-3 definitions, has been used to screen, diagnose, and treat sepsis since September 26, 2017. The system consists of a quick sequential organ-failure assessment (qSOFA) at arrival, determination of the presence or absence of infection, calculation of the sequential organ-failure assessment (SOFA) score, and essential management according to the 2016 Surviving Sepsis Campaign (SSC) guidelines. This study complied with the provisions of the Declaration of Helsinki and was approved by the institutional review board (IRB) of Korea University Medical Center (IRB No. 2017AS0415).

### Study population

During the study period, patients were diagnosed with sepsis if they had an initial positive qSOFA score, the presence of infection, and a SOFA score ≥ 2. Among these patients, we included only adults (≥ 18 years old) who provided written informed consent for the acquisition of biomarker samples. We obtained written informed consent from the legal representatives of patients who were unable to provide consent to participate in the study due to decreased mental capacity. Patients were excluded if they or their legal representatives did not consent to participate or if they presented with cardiac arrest at arrival. Forty-five patients shown to meet initial SIRS criteria but not to have sepsis at a routine check-up (i.e., radiology, blood and urine tests, vital signs, and medical history) in ED were enrolled and served as the control group.

Following data collection, two infectious disease specialists and one emergency attending physician independently reviewed the medical records and clinical data of the enrolled patients to determine sepsis severity and then re-classified enrolled patients into no sepsis, sepsis, and septic shock groups using Sepsis-3 definitions. Light’s kappa value for the three raters (i.e., the average kappa value across all rater pairs) was 0.837.

### Definitions

The Sepsis-3 diagnostic criteria for sepsis include a 2 point or greater increase in SOFA score due to current infection [1, 4]. The qSOFA score is a prompt bedside method that can identify patients with suspected infection who are at greater risk of a poor outcome outside the ICU [1]. The score ranges from 0 to 3 using three criteria that are each assigned one point: low blood pressure (systolic blood pressure ≤ 100 mmHg), high respiratory rate (≥ 22 breaths per min), or altered mentation (Glasgow coma scale score < 15). A positive qSOFA score implies the presence of 2 or more qSOFA signs near the onset of infection. Although qSOFA criteria are only moderately sensitive regarding sepsis, we adopted positive qSOFA scores as inclusion criteria for the present study. The criteria for septic shock include the requirement of a vasopressor to maintain a mean arterial pressure of 65 mmHg and a serum lactate level greater than 2 mmol/L despite adequate fluid resuscitation [4]. In accordance with SSC guidelines, serum lactate levels were measured in all patients. Sepsis severity was assessed by both the SOFA score and an acute physiology and chronic health evaluation II (APACHE II) score [33, 34].

### Sampling for biomarkers and clinical data collection

All blood samples for initial IL-6, PTX3, and PCT measurements were obtained within 6 h of the clinical diagnosis of sepsis or septic shock. Whole blood was collected in serum-separating tubes. Serum was separated, and aliquots were frozen at − 80 °C until analysis. Serum biomarker levels were measured in duplicate in samples kept on ice prior to measurement. Serum IL-6 and PTX3 levels were measured using commercially available enzyme-linked immunosorbent assays (ELISA, R&D Systems, Minneapolis, MN, USA). IL-6 inter- and intra-assay variabilities were 4.5 ± 1.7% and 2.6 ± 1.4%, respectively, and those of PTX3 were 5.1 ± 1.1% and 3.9 ± 0.4%, respectively. PCT levels in serum were measured using reagents from Thermo Fisher Scientific (Thermo Fisher Scientific Clinical Diagnostics, BRAHMS GmbH, Hennigsdorf, Germany). CRP levels were measured by an immunoturbidimetric assay using a Modular P800 automatic analyzer (Roche Diagnostics GmbH, Mannheim, Germany).

Vital signs (blood pressure, heart rate, respiratory rate, and body temperature), routine laboratory test results (creatinine, bilirubin, platelet count, CRP, PCT, hemoglobin, hematocrit, sodium, potassium, urea, lactate, white blood cell count, and blood culture), biomarker measurement (PTX3 and IL-6), blood gas analysis (pH, PaO_2_, PaCO_2_, bicarbonate, and base excess), Glasgow coma scale (GCS) scores, and personal information (age, sex, body weight, and prior medical history) were collected and documented.

### Statistical analysis

We performed statistical analysis using PASW Statistics for Windows version 18.0 (SPSS Inc., Chicago, IL, USA) and Medcalc Statistical Software version 18.5 (Medcalc Software bvba, Ostend, Belgium). Comparisons between two groups were performed using Student’s *t*-test. For more than two groups, quantitative variables were compared using one-way analysis of variance or the Kruskal–Wallis test according to the distribution normality. For quantitative variables, Student’s *t*-test or the Mann–Whitney *U*-test were applied depending on data distribution. Qualitative variables were analyzed using a 2 × 2 contingency table and chi-squared test or Fisher’s exact test as appropriate. Quantitative variables are presented as the mean ± standard error of the mean or as median and interquartile ranges (IQRs), according to data distribution. The accuracy of IL-6, PTX3, and PCT in differentiating sepsis and septic shock patients from controls was assessed using receiver operating characteristic curves. In this method, a perfect biomarker has 100% sensitivity, shows no false positives (100% specificity), and produces an area under the curve (AUC) of 1.0, while a biomarker with no diagnostic value has an AUC of 0.5. We used Youden’s index with the highest sum of sensitivity and specificity to determine the optimal cut-off value for differentiation. Correlations between the levels of IL-6, PTX3, PCT, CRP, and lactate; SOFA score; and APACHE II score were analyzed using Spearman’s rank test. Kaplan–Meier curve analysis and a log-rank test were performed to assess the cumulative survival rate and compare the survival curves of groups with lower IL-6 or PTX3 levels with those of the higher-level groups. Univariate and multivariate Cox regression analyses were performed to evaluate the risk factors for 28-day mortality.

## Results

### Patients’ demographics

**Fig. 1:**
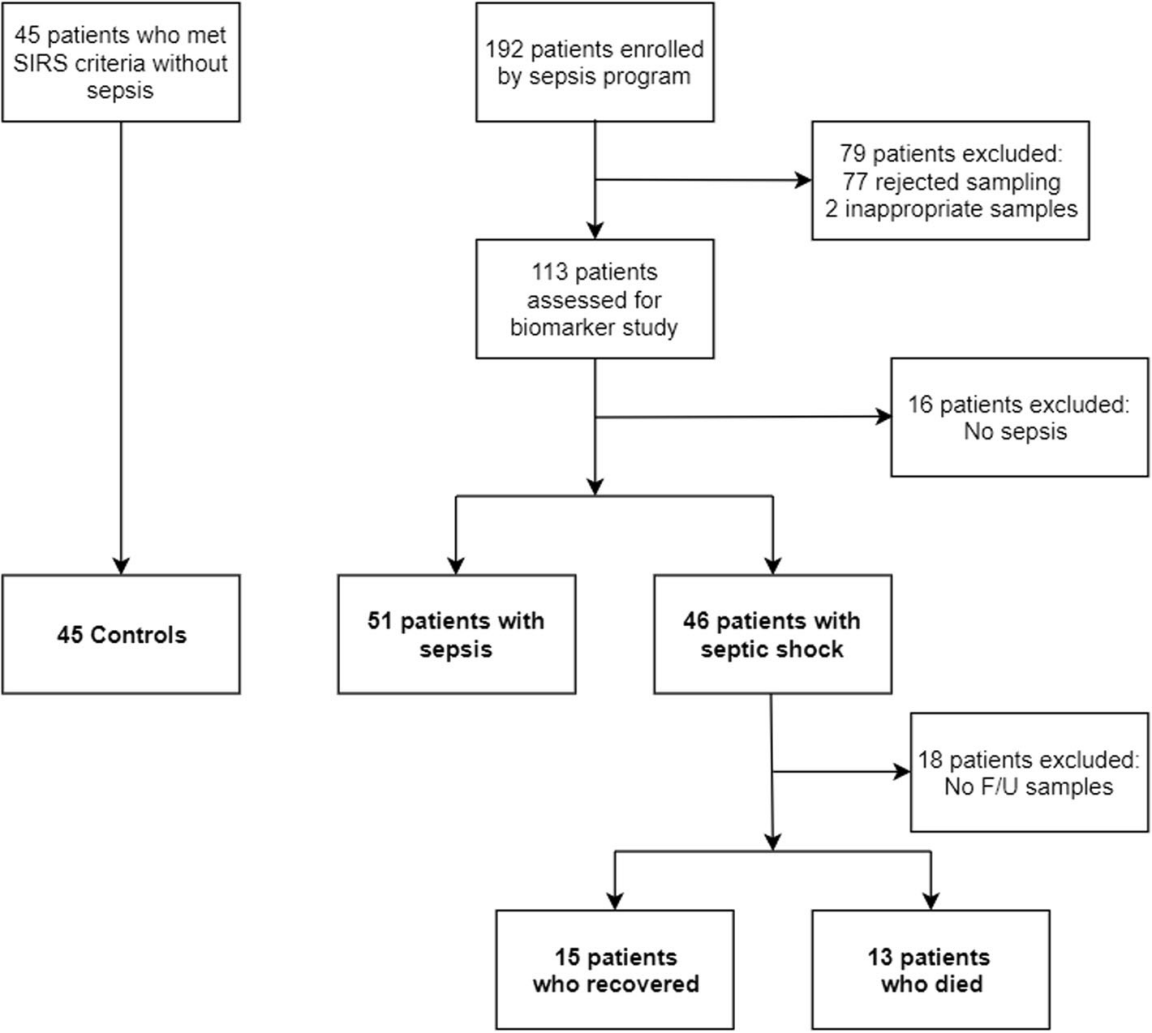
Flow Chart of the Study Population. F/U, follow-up

A flow chart of the study population is presented in Fig. 1. Of the 192 patients clinically diagnosed with sepsis and septic shock, 79 were excluded either due to refusal of study participation (*n* = 77) or inadequate samples (*n* = 2). Therefore, 113 blood samples were initially eligible for biomarker measurements (64 for sepsis and 49 for septic shock). After retrospective re-evaluation, 16 subjects in the initial sepsis group were excluded because they did not meet the criteria for sepsis (i.e., no evidence of infection). The final diagnoses of these 16 subjects were congestive heart failure (*n* = 3), pulmonary thromboembolism (*n* = 2), acute kidney injury (n = 2), a hyperosmolar hyperglycemic state (n = 2), hepatorenal syndrome (n = 2), chronic obstructive pulmonary disease (n = 2), and others (n = 3). After retrospective re-evaluation, three subjects in the initial septic shock group were recategorized to the sepsis group because they did not meet the criteria for septic shock. The final patient populations were as follows: 51 with sepsis, 46 with septic shock, and 45 as controls. Overall, 142 subjects were enrolled in the present study. From the 46 septic shock patients admitted to our institution, follow-up samples were obtained from 28 patients within 24 h of discharge to measure IL-6 and PTX3, 15 of whom recovered, and 13 died. The remaining 18 patients were excluded due to refusal to provide a blood sample, transfer to other institutions, sudden death, or undetermined outcomes (still on admission). Baseline characteristics of the study population are shown in Table 1. The most common infection sites were the respiratory (64.9%) and genitourinary systems (33.0%).

**Table 1:** Baseline Characteristics of the Study Population

Variable	Total patients (*n* = 97)	Sepsis (*n* = 51)	Septic shock (*n* = 46)	Controls(*n* = 45)
Age, median (range)	75 (42–98)	76 (42–98)	74 (42–96)	68 (37–81)
Sex, n (%)				
Male	54 (56)	28 (55)	26 (57)	25 (56)
Female	43 (44)	23 (45)	20 (43)	20 (44)
Infection site, *n* (%)				
Respiratory	63 (64.9)	33 (64.7)	30 (65.2)	–
Genitourinary	32 (33.0)	19 (37.3)	13 (28.3)	–
Cardiovascular	3 (3.1)	0 (0.0)	3 (6.5)	–
Gastrointestinal	2 (2.1)	0 (0.0)	2 (4.3)	–
Musculoskeletal	2 (2.1)	1 (2.0)	1 (2.2)	–
Central nervous	1 (1.0)	0 (0.0)	1 (2.2)	–
Hepatobiliary	1 (1.0)	0 (0.0)	1 (2.2)	–
Skin and soft tissue	1 (1.0)	1 (2.0)	0 (0.0)	–
Unknown	5 (5.2)	2 (3.9)	3 (6.5)	–
Underlying disease				
Coronary artery disease	11 (11.3)	5 (9.8)	6 (13.0)	–
Malignancy	10 (10.3)	4 (7.8)	6 (13.0)	–
Rheumatic disease	4 (4.1)	2 (3.9)	2 (4.3)	–
SOFA score, median (IQR)	8 (4–11)	6 (3–9)	10 (6–13)	–
APACHE II score, median (IQR)	21 (13–30)	18 (10–27)	25 (16–35)	–
Laboratory value,median (IQR) or mean ± SEM				
Procalcitonin (ng/mL)	1.6 (0.5–10.7)	0.3 (0.2–1.2)	3.4 (1.6–20.3)	
CRP (mg/dL)	10 (6–20)	10 (5–20)	11 (7–21)	
Lactate (mmol/L)	3.6 (2.6–4.6)	1.9 (1.1–2.8)	5.5 (3.6–7.5)	
Creatinine (mg/dL)	2.5 ± 0.2	2.1 ± 0.2	2.9 ± 0.2	
Bilirubin (mg/dL)	2.2 ± 0.4	1.7 ± 0.3	2.8 ± 0.5	
Platelet (× 1000/μL)	203 ± 12.4	251 ± 14.3	153 ± 10.6	
Positive blood cultures, n (%)	75 (77.3)	35 (68.6)	40 (87.0)	–
ICU days, median (IQR)	9 (5–14)	8 (4–11)	11 (7–16)	–
Length of stay, median (IQR)	13 (8–18)	11 (7–16)	15 (9–19)	–

*SOFA* sequential organ-failure assessment, *IQR* interquartile range, *APACHE* acute physiology and chronic health evaluation, *CRP* C-reactive protein, *SEM* standard error of the mean, *ICU* intensive care unit

### Correlations with other biomarkers and severity score

IL-6 levels showed positive correlations with PTX3 (rho = 0.802, *P* <  0.001), lactate (rho = 0.798, *P* <  0.001), PCT (rho = 0.752, *P* <  0.001), CRP (rho = 0.476, P <  0.001), SOFA score (rho = 0.421, *P* < 0.001), and APACHE II score (rho = 0.407, *P* < 0.001) by Spearman’s rank analysis.

### Diagnostic value of biomarkers

**Fig. 2:**
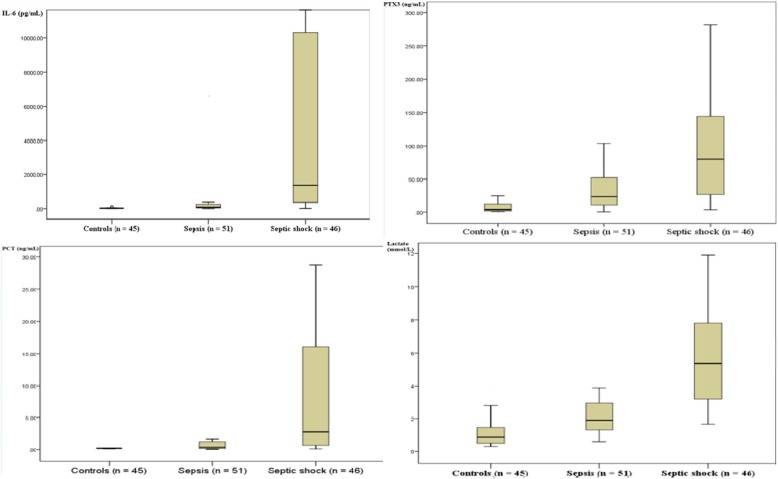
Interleukin-6, pentraxin 3, procalcitonin, and lactate levels in sepsis and septic shock patients. Patients were diagnosed in the emergency department according to Sepsis-3 definitions. CRP, C-reactive protein

The median IL-6 values (IQR) in the control, sepsis, and septic shock groups were 23.6 (11.2–43.5), 89.9 (45.2–272.6), and 1378.6 (256.4–11,062.1) pg/mL, respectively (Fig. 2); those for PTX3 were 4 (2–13), 25 (10–51), and 74 (26–147) ng/mL, respectively; those for PCT were 0.2 (0.1–0.23), 0.3 (0.1–1.1), and 3.4 (1.3–20.1) ng/mL, respectively; and those for lactate were 0.9 (0.5–1.5), 1.9 (0.9–2.9), and 5.5 (3.0–8.1) mmol/L, respectively. The three groups showed significant differences in the levels of these four biomarkers, as determined by Kruskal–Wallis and post hoc tests (*P* < 0.001). The median CR*P* values (IQR) in the control, sepsis, and septic shock groups were 3.6 (2.0–5.2), 9.9 (4.9–20.2), and 10.5 (7.3–21.0) mg/dL, respectively. There were significant differences between the control and other groups (*P* < 0.001); however, no significant difference was found between the sepsis and septic shock groups (*P* = 0.45).

**Fig. 3:**
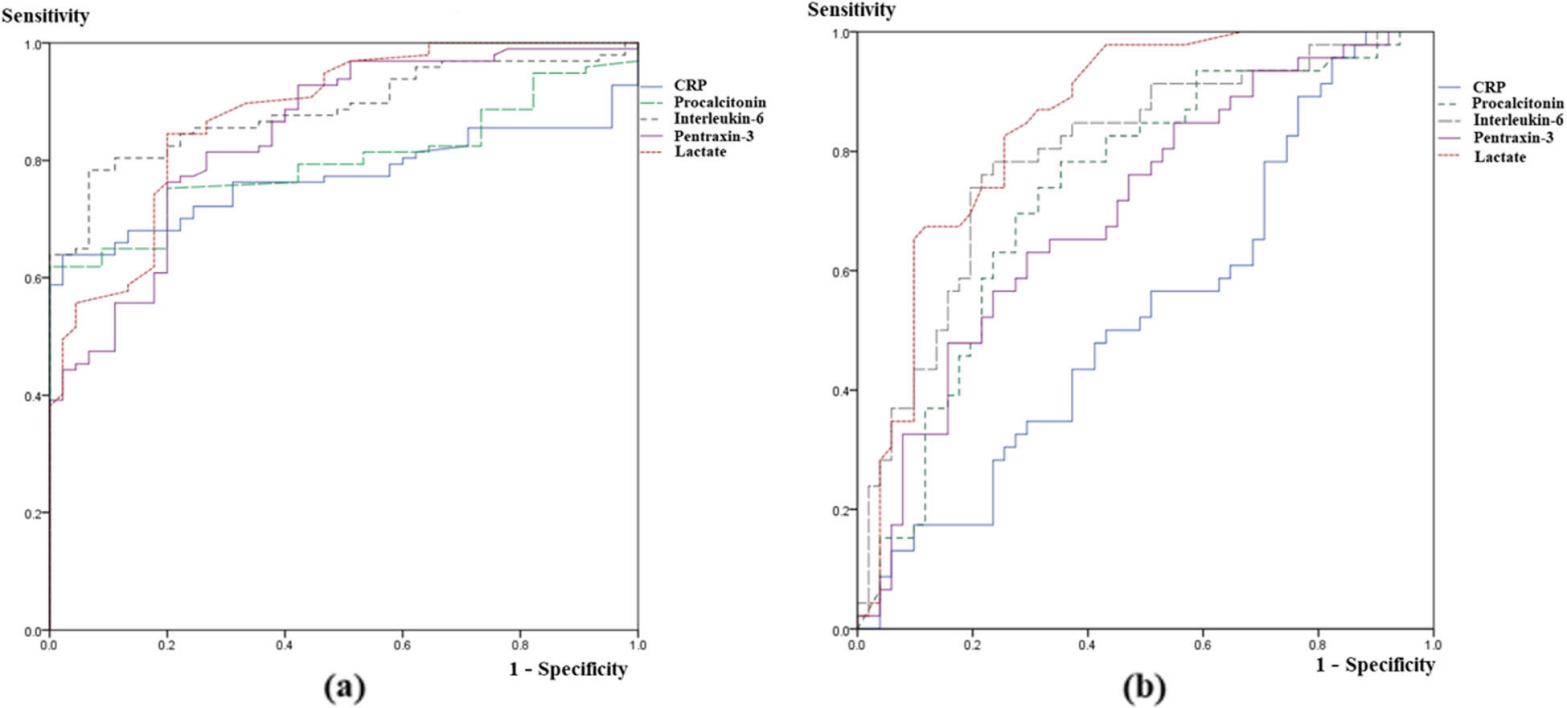
Receiver operating characteristic curves for distinguishing sepsis or septic shock. Sepsis (**a**) and septic shock (**b**) are variously discriminated by interleukin-6, pentraxin 3, lactate, and procalcitonin levels measured in the emergency department

The AUC of IL-6 to discriminate sepsis from the control group was 0.89 (95% confidence interval [CI], 0.97–1.00; *P* < 0.001), 0.84 for PTX3 (95% CI, 0.95–0.99; *P* < 0.001), 0.80 for PCT (95% CI, 0.86–0.96; P < 0.001), and 0.77 for CRP (95% CI, 0.71–0.91; *P* < 0.001) (Table 2 and Fig. 3). The optimal cut-off value to discriminate sepsis from controls was 52.60 pg/mL for IL-6 (sensitivity, 97.0%; specificity, 97.2%; *P* < 0.001) (Table 3) and 15.10 ng/mL for PTX3 (sensitivity, 92.6%; specificity, 97.4%; *P* < 0.001).

**Table 2:** Comparisons of the Discriminating Capacities between Biomarkers Presented as Areas Under the Curve (95% CI)

Severity	Interleukin-6	Pentraxin 3	Procalcitonin	Lactate	C-reactive protein
**≥Sepsis** (*n* = 97)	0.89 (0.83–0.94) *P* < 0.001	0.84 (0.78–0.91) *P* < 0.001	0.80 (0.73–0.87) *P* < 0.001	0.88 (0.82–0.94) *P* < 0.001	0.77 (0.70–0.85) *P* < 0.001
Septic shock (*n* = 46)	0.80 (0.71–0.89) *P* < 0.001	0.70 (0.60–0.81) *P* = 0.001	0.73 (0.63–0.83) *P* < 0.001	0.85 (0.77–0.93) *P* < 0.001	0.53 (0.42–0.65) *P* = 0.603

*CI* confidence interval

**Table 3:** Diagnostic Value of Interleukin-6 and Pentraxin 3 for Patients with Sepsis and Septic Shock

Biomarker	Severity	AUC (95% CI)	Cut-off value	Sensitivity (%)	Specificity (%)	*P* value
IL-6	Sepsis	0.89 (0.83–0.94)	52.60	80.4	88.9	< 0.001
(pg/mL)	Septic shock	0.80 (0.71–0.89)	348.92	76.1	78.4	< 0.001
PTX3	Sepsis	0.84 (0.78–0.91)	15.10	76.3	80.0	< 0.001
(ng/mL)	Septic shock	0.70 (0.60–0.81)	58.28	56.5	76.5	0.001

*AUC* area under the curve, *CI* confidence interval, *IL-6* interleukin-6, *PTX3* pentraxin 3

The AUC to discriminate septic shock was 0.80 for IL-6 (95% CI, 0.71–0.89; *P* < 0.001), 0.70 for PTX3 (95% CI, 0.60–0.81; P < 0.001), 0.73 for PCT (95% CI, 0.63–0.83; *P* < 0.001), and 0.53 for CRP (95% CI, 0.42–0.65; *P* = 0.603) (Table 2 and Fig. 3). The optimal cut-off value to discriminate septic shock was 348.92 pg/mL for IL-6 (91.8% sensitivity, 63.2% specificity, P < 0.001) (Table 3) and 58.28 ng/mL for PTX3 (93.2% sensitivity, 60.7% specificity, P < 0.001).

### Prognostic value of biomarkers

Univariate and multivariate Cox regression analysis results for the risk factors of 28-day mortality are shown in Table 4. The univariate analysis determined that the significant risk factors for 28-day mortality were IL-6, PTX3, lactate, SOFA and APACHE II scores, and septic shock. The significant risk factors determined by multivariate analysis were IL-6 (hazard ratio [HR], 1.0004; 95% CI, 1.0003–1.0005; *P* = 0.024), SOFA score (HR, 1.128; 95% CI, 1.030–1.211; *P* = 0.011), and lactate (HR, 1.135; 95% CI, 1.033–1.247; *P* = 0.009).

**Table 4:** Univariate and Multivariate Cox Proportional Models of Risk Factors for 28-day Mortality

Variable	HR (95% CI)	*P* value	Adjusted HR (95% CI)	*P* value
Age	1.023 (0.994–1.052)	0.120		
Male sex	1.029 (0.535–1.978)	0.932		
SOFA score	1.206 (1.076–1.353)	0.001	1.128 (1.030–1.211)	0.011
APACHE II score	1.198 (1.068–1.346)	0.001	1.031 (0.898–1.187)	0.231
Pentraxin 3	1.005 (1.001–1.009)	0.031	1.003 (0.998–1.008)	0.095
Interleukin 6	1.001 (1.000–1.002)	0.017	1.001 (1.000–1.002)	0.024
Procalcitonin	0.995 (0.981–1.009)	0.481		
Lactate	1.167 (1.068–1.275)	0.001	1.135 (1.033–1.247)	0.009
CRP	1.011 (0.978–1.045)	0.525		
Septic shock	2.657 (1.327–5.317)	0.004	1.249 (0.472–3.302)	0.240

*HR* hazard ratio, *CI* confidence interval, *GCS* Glasgow coma scale, *SOFA* sequential organ-failure assessment, *APACHE* acute physiology and chronic health evaluation, *CRP* C-reactive protein

**Fig. 4:**
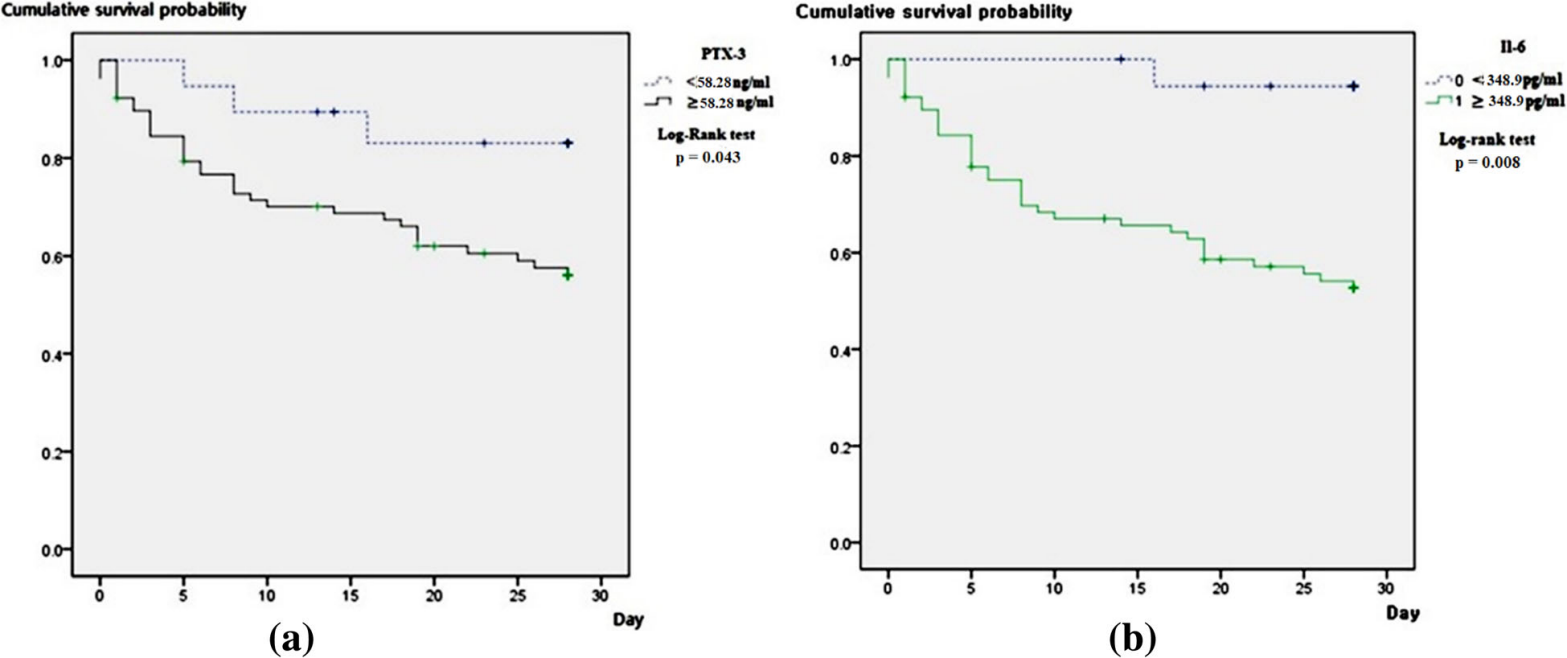
Kaplan-Meier curve of 28-day mortality in patients with sepsis and septic shock. The curve is stratified by the optimal cut-off value of pentraxin 3 (**a**) and interleukin-6 (**b**) to predict septic shock (28-day mortality by pentraxin 3: 14.6% vs. 45.9%; 28-day mortality by interleukin-6: 5.1% vs. 49.1%)

Kaplan–Meier curve analyses and log-rank tests were performed to assess cumulative survival rates and compare 28-day survival curves between the high IL-6 (≥ 348.9 pg/mL) and low IL-6 (< 348.9 pg/mL) groups. The optimal cut-off value of IL-6 to predict septic shock in the present study was 348.9 pg/mL. The survival curve of the high IL-6 group significantly differed from that of the low IL-6 group in log-rank tests (*P* = 0.008) (Fig. 4). Kaplan–Meier curve analyses and log-rank tests were also performed to assess cumulative survival rates and compare the 28-day survival curves between the high PTX3 (≥ 58.28 ng/mL) and low PTX3 (< 58.28 ng/mL) groups. The optimal cut-off value of PTX3 to predict septic shock was 58.28 ng/mL. The survival curve of the high PTX3 group significantly differed from that of the low PTX3 group in log-rank tests (*P* = 0.043) (Fig. 4).

**Fig. 5:**
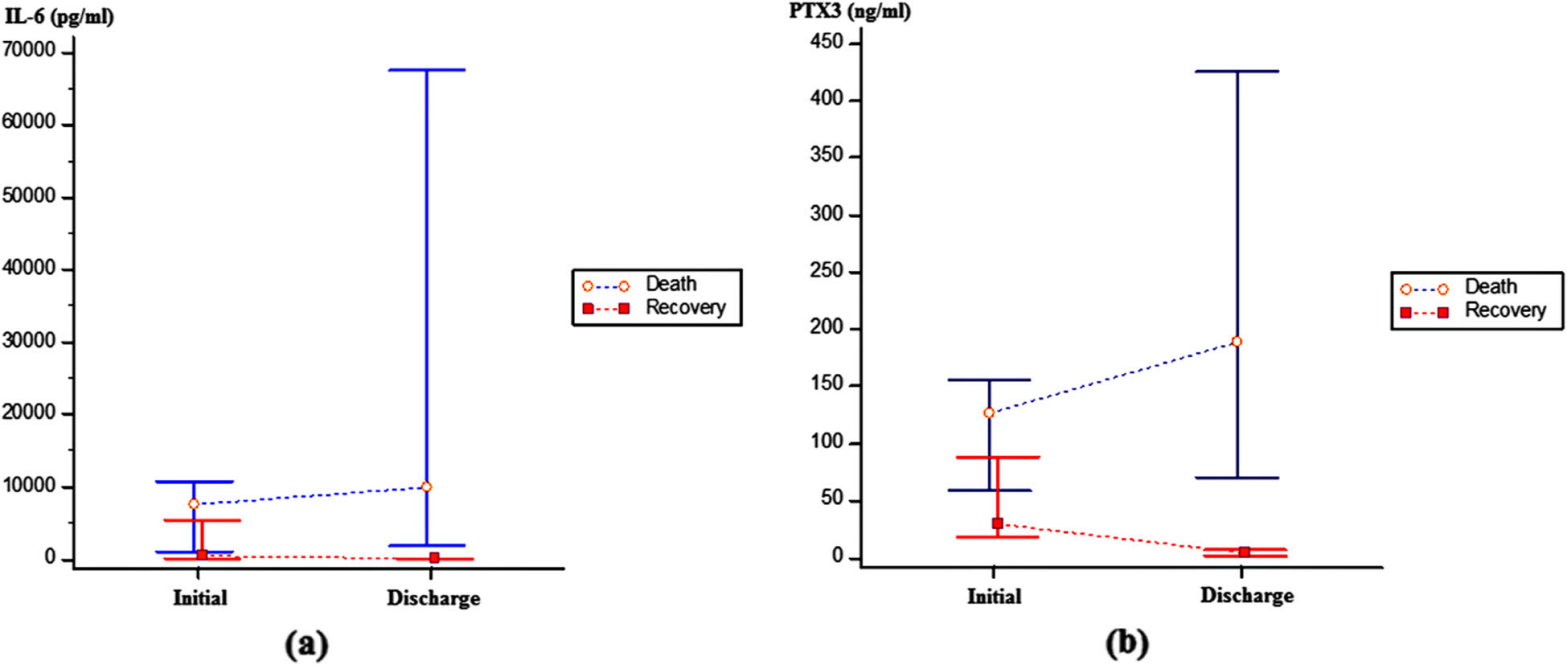
Error bars of initial and follow-up levels of interleukin-6 and pentraxin 3. Error bars are shown for the levels of interleukin-6 (**a**) and pentraxin 3 (**b**) in septic shock patients who died or recovered during admission. Initial and follow-up levels were taken within 6 h of clinical diagnosis and 24 h of discharge, respectively

Among the patients with septic shock at presentation, initial IL-6 levels within 6 h of clinical diagnosis in the recovered survivors (*n* = 15) and non-survivors (*n* = 13) were 444.3 (261.2–5893.5) and 7609.5 (4526.0–12,208.4) pg/mL, respectively (*P* = 0.05), and follow-up IL-6 levels within 24 h of discharge were 21.5 (10.2–51.7) and 9976.5 (4651.2–71,048.3) pg/mL, respectively (*P* < 0.001). Among the same patients, initial PTX3 levels within 6 h of clinical diagnosis were 29 (10–75) and 126 (70–147) ng/mL, respectively (*P* = 0.007) (Fig. 5), and follow-up PTX3 levels within 24 h of discharge were 4 (2–7) and 188 (101–376) ng/mL, respectively (*P* < 0.001. Error bars represent the variability of data in Fig. 5. Both IL-6 and PTX3 levels significantly decreased in the recovered survivors (P < 0.001 and P < 0.001, respectively); however, both levels remained high and even significantly increased in the non-survivors (*P* = 0.03 and *P* = 0.009, respectively).

### Combination of IL-6 and PTX3 to predict severity and mortality

Multivariate logistic regression was performed to model the ability of the biomarker combination (IL-6 and PTX3) to discriminate septic shock patients from sepsis and to predict 28-day mortality among the overall patients. Using the logistic regression equation, the log of probability was converted to the probability of septic shock and 28-day mortality. The AUC values of combined marker score (probability of septic shock) and each biomarker for the discrimination of septic shock are shown in Table 5. The AUC values of combined marker score (probability of 28-day mortality) and each biomarker for the prediction of 28-day mortality are shown in Table 6.

**Table 5:** Discriminating performance of the combined marker (IL-6 + PTX3), IL-6, and PTX3 for septic shock

Biomarkers	AUC (95% CI)	*p*	Cut-off	Sensitivity (%)	Specificity (%)
IL-6 + PTX3	0.806 (0.719–0.894)	< 0.001	52.0 (score)	82.7	71.1
IL-6	0.795 (0.702–0.883)	< 0.001	348.92 (pg/mL)	76.1	78.4
PTX3	0.703 (0.603–0.812)	0.001	58.28 (ng/mL)	56.5	76.5

*AUC* area under the curve, *CI* confidence interval, *IL-6* interleukin-6, *PTX3* pentraxin 3

**Table 6:** Predictive performance of the combined marker (IL-6 + PTX3), IL-6, and PTX3 for 30-day mortality

Biomarkers	AUC (95% CI)	p	Cut-off	Sensitivity (%)	Specificity (%)
IL-6 + PTX3	0.637	0.025	33.1 (score)	63.9	62.3
IL-6	0.674	0.004	263.3	69.4	60.7
PTX3	0.645	0.017	36.0	72.2	59.0

*AUC* area under the curve, *CI* confidence interval, *IL-6* interleukin-6, *PTX3* pentraxin 3

## Discussion

In the present study, we evaluated the diagnostic and prognostic values of IL-6, PTX3, PCT, CRP, and lactate in patients with sepsis and septic shock diagnosed using Sepsis-3 definitions. The results showed that serum IL-6 and PTX3 levels could identify the severity of sepsis (sepsis, septic shock, and controls) with optimal cut-off values. IL-6 had superior diagnostic and prognostic value compared with PTX3, PCT, and CRP. Furthermore, IL-6 was an independent risk factor for 28-day mortality in patients with sepsis and septic shock.

Several previous studies have presented conflicting results regarding the diagnostic value of biomarkers such as IL-6, PTX3, PCT, presepsin, and CRP [12, 16, 18–20, 32, 35, 36]. Reports in which IL-6 was a better diagnostic marker for sepsis than PCT, presepsin, and CRP [20, 35] are consistent with our study. However, some studies have demonstrated that the diagnostic value of PCT was superior to that of IL-6 [16, 19, 36], and others have suggested that the diagnostic value of IL-6 was nearly equal to that of PCT or PTX3 in septic patients [12, 32]. Previous studies that evaluated the clinical value of several biomarkers including IL-6 are shown in Table 7.

**Table 7:** Studies on the Clinical Value of Biomarkers Including Interleukin-6 in Sepsis Patients

Author [reference No.]	Song et al.	Takahashi et al. [20]	Behnes et al. [35]	Hamed et al. [32]	Harbarth et al. [16]	Jekarl et al. [19]	Mat-Nor et al. [36]	Miguel-Bayarri et al. [18]
Year of publication	2019	2016	2014	2017	2001	2013	2016	2011
Definitions	Sepsis-3	Sepsis-2	Sepsis-2	Sepsis-3	Sepsis-2	Sepsis-2	Sepsis-2	Sepsis-2
Setting	ED	ICU	ICU	ICU	ICU	ED	ICU	ICU
Biomarkers	IL-6, PTX3 PCT, CRP	IL-6, PCT, presepsin, CRP	IL-6, PCT, presepsin, CRP	IL-6, PTX3, PCT, CRP	IL-6, PCT, IL-8	IL-6, PCT, CRP	IL-6, PCT	IL-6, PCT, CRP
Case	Sepsis	Severe sepsis, septic shock	Septic shock	Sepsis	Sepsis	Severe sepsis, septic shock	Sepsis	Severe sepsis, septic shock
Control	Healthy control, Patient control	No infection with organ dysfunction	Patient control, SIRS, sepsis, severe sepsis	Healthy control, patient control	SIRS	SIRS, sepsis	SIRS	–
Diagnostic value	IL-6> PTX3> PCT > CRP	IL-6 > PCT > CRP> presepsin	IL-6 > PCT > presepsin > CRP	IL-6 = PTX3 = PCT > CRP	PCT > IL-6 > IL-8	PCT > IL-6 > CRP	PCT > IL-6	–
Most valuable prognostic marker	IL-6	IL-6	–	–	–	IL-6	–	IL-6

*ED* emergency department, *ICU* intensive care unit, *IL-6* interleukin-6, *PTX3* pentraxin 3, *PCT* procalcitonin, *CRP* C-reactive protein

A recent study reported that serum IL-6 levels had the highest diagnostic value for infection in patients with organ dysfunction compared with PCT and CRP levels [20]. Another study reported that serum IL-6 levels had the highest diagnostic value for septic shock compared with PCT, presepsin, and CRP [35]. These results are in agreement with our result that IL-6 was superior to PTX3, PCT, and CRP in diagnostic value for sepsis and septic shock. In contrast to our study, some have reported that PCT is superior to IL-6 for diagnosing severe sepsis and septic shock [16, 19, 36, 37]. We postulate that this discrepancy was caused by not only different settings (ED versus ICU), but also different severity levels or definitions of sepsis among the study populations (Sepsis-2 versus Sepsis-3). According to Hamed et al., IL-6 has diagnostic value that is comparable, but not superior, to PTX3 and PCT in patients with sepsis and septic shock diagnosed using Sepsis-3 definitions [32]. A recent meta-analysis reported that IL-6 and PCT had similar diagnostic values that were higher than that of CRP [12].

In line with the current study, several studies showed that, among various inflammatory biomarkers, IL-6 is the most valuable for predicting outcomes [18–20, 36]. These studies demonstrated that IL-6 is an independent predictor of in-hospital mortality. Another study reported that IL-6 exhibits superior kinetics when monitoring the effectiveness of antibiotic treatments [19] and suggested that clinicians can use IL-6 as a prognostic marker in sepsis. In the present study, IL-6 levels in the initial blood samples obtained from patients with septic shock significantly decreased in the recovery group but increased among the death group, which suggests that IL-6 levels can be used to monitor the effectiveness of treatment for septic shock. Another study showed that IL-6 levels in septic shock did not fall near normal levels within the first 24 h of treatment and instead remained high until clinical recovery [15]. In the present study, because the follow-up IL-6 levels were measured within 24 h of discharge (recovery or death), direct comparison with the previous study may be difficult. Nevertheless, we speculate that combining follow-up IL-6 levels with initial levels could be of additional value for predicting mortality in patients with septic shock.

PTX3 was proposed as a diagnostic and prognostic marker for sepsis [10, 23–28, 30, 31, 35]. In a recent systemic review and meta-analysis, PTX3 was reported as a marker of sepsis severity and predictor of mortality [31]; however, these results were drawn from sepsis diagnoses determined by the past Sepsis-2 definitions. In a recent study, PTX3 distinguished sepsis and septic shock from controls, which corresponds to uniform cut-off levels in accordance with Sepsis-3 definitions [32]. This study enrolled patients from the ICU and did not assess the prognostic value of PTX3 in sepsis. According to Raija et al., high PTX3 levels at hospital admission predict severe sepsis and case fatality in patients with suspected infection [23]. However, in the current study, PTX3 was not a significant predictor of 28-day mortality in multivariate Cox regression analysis.

Our study showed that groups with high IL-6 and PTX3 levels have higher 28-day mortality than those with low IL-6 and PTX3, and these results were consistent with those reported by some other studies. Previous studies revealed that IL-6 is an independent predictor for mortality [18–20, 36]; one cohort study suggested that PTX3 levels at admission can predict 28-day mortality in a community-based hospital [38], and another study reported that PTX3 can predict 30-day and 6-month mortality in patients with sepsis and septic shock during intensive care treatment [39]. A prior study reported that PTX3 levels were below 2 ng/mL in normal healthy persons [40]. In patients with bacteremia, increased PTX3 levels occur in the acute phase of infection and normalize on recovery [41]; we also found that PTX3 levels in patients with septic shock were initially high but normalized on recovery.

We found that the lactate level was an independent predictor of 28-day mortality in patients with sepsis and septic shock, although this marker was not the main focus of the current study. Some studies have reported that lactate levels can predict mortality in severe sepsis [18], and others have demonstrated that lactate is a significant prognostic marker that reflects hypoperfusion, particularly in critically ill patients [42, 43]. Although lactate is not an inflammatory cytokine, it can be used as a valuable prognostic marker in sepsis and septic shock.

The present study has some advantages. First, we performed a prospective controlled study of biomarkers among ED patients with sepsis and septic shock. Second, the research was conducted in accordance with the latest Sepsis-3 definitions. Third, both diagnostic and prognostic values of biomarkers were assessed. To the best of our knowledge, this is the first study to examine both the diagnostic and prognostic values of IL-6, PTX3, PCT, CRP, and lactate in sepsis and septic shock according to the Sepsis-3 definitions.

This study also had some limitations in that it was conducted in a single center at a tertiary referral teaching hospital, and we did not include patients who had infections but failed to meet the sepsis criteria. If these patients were included in the control group, the cut-off value for sepsis and septic shock may have increased. Further, some of the enrolled patients were transferred to our institution with prior sepsis management, including antibiotics, fluids, or vasopressors.

## Conclusions

IL-6 and PTX3 can be used as both diagnostic and prognostic biomarkers for sepsis and septic shock diagnosed in accordance with the Sepsis-3 definitions. Overall, IL-6 was superior to PTX3 and PCT in both diagnostic and prognostic value for sepsis and septic shock. The results of this study should be verified by a prospective controlled multi-center study.

## Data Availability

The datasets supporting the conclusions of this article are available from the corresponding author on reasonable request.

## Abbreviations

APACHE: Acute physiology and chronic health evaluation
AUC: Area under the curve;
CI: Confidence interval
CRP: C-reactive protein
ED: Emergency department
ICU: Intensive care unit
IL-6: Interleukin-6
IQR: Interquartile range.
PCT: Procalcitonin;
PTX3: Pentraxin 3
qSOFA: quick Sequential organ-failure assessment
SEM: Standard error of the mean
